# Enhancement of Vaccinia Virus Based Oncolysis with Histone Deacetylase Inhibitors

**DOI:** 10.1371/journal.pone.0014462

**Published:** 2010-12-30

**Authors:** Heather MacTavish, Jean-Simon Diallo, Baocheng Huang, Marianne Stanford, Fabrice Le Boeuf, Naomi De Silva, Julie Cox, John Graydon Simmons, Tanya Guimond, Theresa Falls, J. Andrea McCart, Harry Atkins, Caroline Breitbach, David Kirn, Stephen Thorne, John C. Bell

**Affiliations:** 1 Center for Cancer Therapeutics, Ottawa Hospital Research Institute, Ottawa, Ontario, Canada; 2 University of Ottawa, Ottawa, Ontario, Canada; 3 Departments of Surgery and Immunology, University of Pittsburgh Cancer Institute, Hillman Cancer Center, University of Pittsburgh, Pittsburgh, Pennsylvania, United States of America; 4 Jennerex Biotherapeutics, Ltd., San Francisco, California, United States of America; 5 Division of Experimental Therapeutics, Toronto General Research Institute, Toronto, Ontario, Canada; University of Hong Kong, Hong Kong

## Abstract

Histone deacetylase inhibitors (HDI) dampen cellular innate immune response by decreasing interferon production and have been shown to increase the growth of vesicular stomatitis virus and HSV. As attenuated tumour-selective oncolytic vaccinia viruses (VV) are already undergoing clinical evaluation, the goal of this study is to determine whether HDI can also enhance the potency of these poxviruses in infection-resistant cancer cell lines. Multiple HDIs were tested and Trichostatin A (TSA) was found to potently enhance the spread and replication of a tumour selective vaccinia virus in several infection-resistant cancer cell lines. TSA significantly decreased the number of lung metastases in a syngeneic B16F10LacZ lung metastasis model yet did not increase the replication of vaccinia in normal tissues. The combination of TSA and VV increased survival of mice harbouring human HCT116 colon tumour xenografts as compared to mice treated with either agent alone. We conclude that TSA can selectively and effectively enhance the replication and spread of oncolytic vaccinia virus in cancer cells.

## Introduction

As biological tumour killing machines, oncolytic viruses (OVs) often display an array of anti-cancer activities including direct tumour lysis, immune cell recruitment and anti-vascular activity [1], [2]. In order to safely implement OVs in the clinic it is critical to restrict their replication and activity to tumours. To date, this has been achieved in part by the engineering or selection of virus variants that have mutations or deletions in viral virulence genes. The proteins encoded by virulence genes often attack or antagonize normal cellular anti-viral programs facilitating the invasion and ultimate destruction of the infected cell. Since OVs have impaired virulence genes they are unable to productively infect normal cells, however, since tumour cells frequently have acquired defects in anti-viral signaling pathways, they remain uniquely sensitive to OV infection and killing. One signaling pathway that is defective in a large proportion of cancer cells (∼70–75%) is the interferon (IFN) pathway, which mediates the first line of cellular anti-viral response [3], [4], [5], [6], [7]. However we and others have shown that the extent of interferon non-responsiveness is variable in tumour cell lines and patient tumour explants and this may lead to less than optimal therapeutic benefit from some OVs [2], [8], [9].

Vaccinia virus (VV) has many of the biological properties that an ideal oncolytic or cancer killing virus should have. It has an extensive safety history in humans, a large cloning capacity for insertion of therapeutic transgene payloads, is active as a systemic agent, lacks any known genotoxic activity and expresses a sophisticated array of immune modulating genes that can be exploited for therapeutic benefit [1]. A Phase I trial of an oncolytic vaccinia virus JX-594 demonstrated acceptable safety and promising anti-cancer activity in patients with advanced liver tumours [10].

Vaccinia encodes close to two hundred genes, some of which are now known to be redundant for growth in tumour cells [1], [11], [12]. For example VV mutants with deletions in the thymidine kinase gene (TK) and/or the vaccinia growth factor gene (VGF) are well advanced in pre-clinical and clinical studies [1], [10], [13], [14]. These mutants grow selectively in cancer cells in which high levels of cellular TK and constitutively activated EGFR/Ras pathway signaling complements the loss of the viral gene products [12]. Another vaccinia gene that can be manipulated to enhance virus selectivity for cancer cells is B18R which encodes a soluble mimetic of the type-1 interferon receptor. When produced and secreted from VV infected cells the B18 protein locally blunts the cellular interferon response by sequestering interferon produced by the infected cell [15], [16], [17]. Previously, we have shown that a VV strain with an engineered deletion of the B18R gene is more rapidly cleared from normal tissues than the parental strain while remaining active within tumours [18]. A natural truncation of the B18R gene of the clinical vaccinia candidate JX-594, has been shown by others to have reduced ability to antagonize interferon activity [16] and this likely contributes to its acceptable safety profile in humans [10].

As mentioned above, while defects in innate anti-viral responses are common in malignant cells the extent of the defect is variable and can affect the growth of OVs in tumours. In an earlier study we showed that a Histone Deacetylase Inhibitor (HDI) can specifically enhance the growth of an interferon sensitive version of vesicular stomatitis virus (VSVΔ51) in tumour cells. HDIs block the activity of histone deacetylases (HDACs), leading to increased acetylation of histones and other proteins [4], [19], [20], [21] and importantly inhibit the ability of tumour cells to mount a productive anti-viral response [22], [23], [24]. In the current study we set out to examine the ability of a panel of HDIs to augment oncolytic activity of vaccinia virus. We present evidence that the growth of vaccinia virus is most potently and selectively enhanced in tumour cells both *in vitro* and *in vivo* by the HDI trichostatin A.

## Results

### Trichostatin A is a potent enhancer of vaccinia virus spread

A number of HDIs are in clinical development and we assessed a panel of candidates for their ability to enhance vaccinia virus replication and spread in tumour cells. 4T1 murine breast cancer cells were pre-treated with individual HDIs over a range of concentrations then challenged with a Green Fluorescent Protein (GFP)-expressing vaccinia virus (herein referred to as VVdd) [12], at a multiplicity of infection (MOI) of 0.1 plaque forming units (pfu) per cell. The percentage of GFP positive cells visible (indicating active virus replication) following 120 h incubation was subsequently assessed from fluorescence microscopy images quantified using image analysis software. Each condition was related to percent GFP-positive area in the vehicle control. Table 1 shows that several HDIs enhance the spread of VVdd albeit to varying extents. Overall, we found that Valproic Acid, SBHA, M344 and Trichostatin A (TSA) elicited the greatest response however clearly TSA was the most potent enhancer of VVdd replication (Table 1). We confirmed by western blot analysis that all the HDIs were able to affect histone acetylation in treated cells and the extent of histone 4 acetylation generally corresponded with the ability of the HDI to enhance VVdd growth. Since TSA seemed to be the most potent HDI in these studies we chose to test it in a variety of *in vitro* an *in vivo* models.

**Table 1:** TSA is a potent enhancer of vaccinia virus spread.

HDI	Maximum effect	Effective Dose ( µM)	Level of Acetylated H4
SAHA	++	0.8	33.7
MS-275	+	1.6	26.6
Oxamflatin	++	2.5	28.0
Apicidin	+	0.25	26.7
SBHA	+++	25	50.8
Scriptaid	++	0.5	16.0
CHAHA	+	0.6	4.7
Valproic Acid	+++	1250	1.5
M344	+++	0.6	66.2
***TSA***	***+++***	***0.08***	***52.7***

4T1 cells were plated in 96-well plates then pre-treated with a concentration gradient of the indicated drugs. DMSO was used as a control. Following pre-treatment with drugs, cells were challenged with VVdd-GFP at an MOI of 0.1. After 120 h incubation period, fluorescence pictures were taken of each well, spanning the entire well-surface. Green fluorescence, indicating vaccinia spread, was measured using an image analysis software (Image J, NIH) and reported as a fold change in GFP-positive comparison to control (average of triplicate). + indicates increase in spread <2-fold, ++ indicates between 2 and 3-fold increase, +++ indicates >3-fold increase. TSA was the most active compound at the lowest dose tested. 10 cm plates of confluent 4T1 cells were treated with indicated HDI and samples were probed for hyper-acetylated histone H4 by Western Blot and reported as level of acetylated H4 relative to the untreated control.

### TSA enhances the spread of VV strains specifically in cancer cells

**Figure 1:**
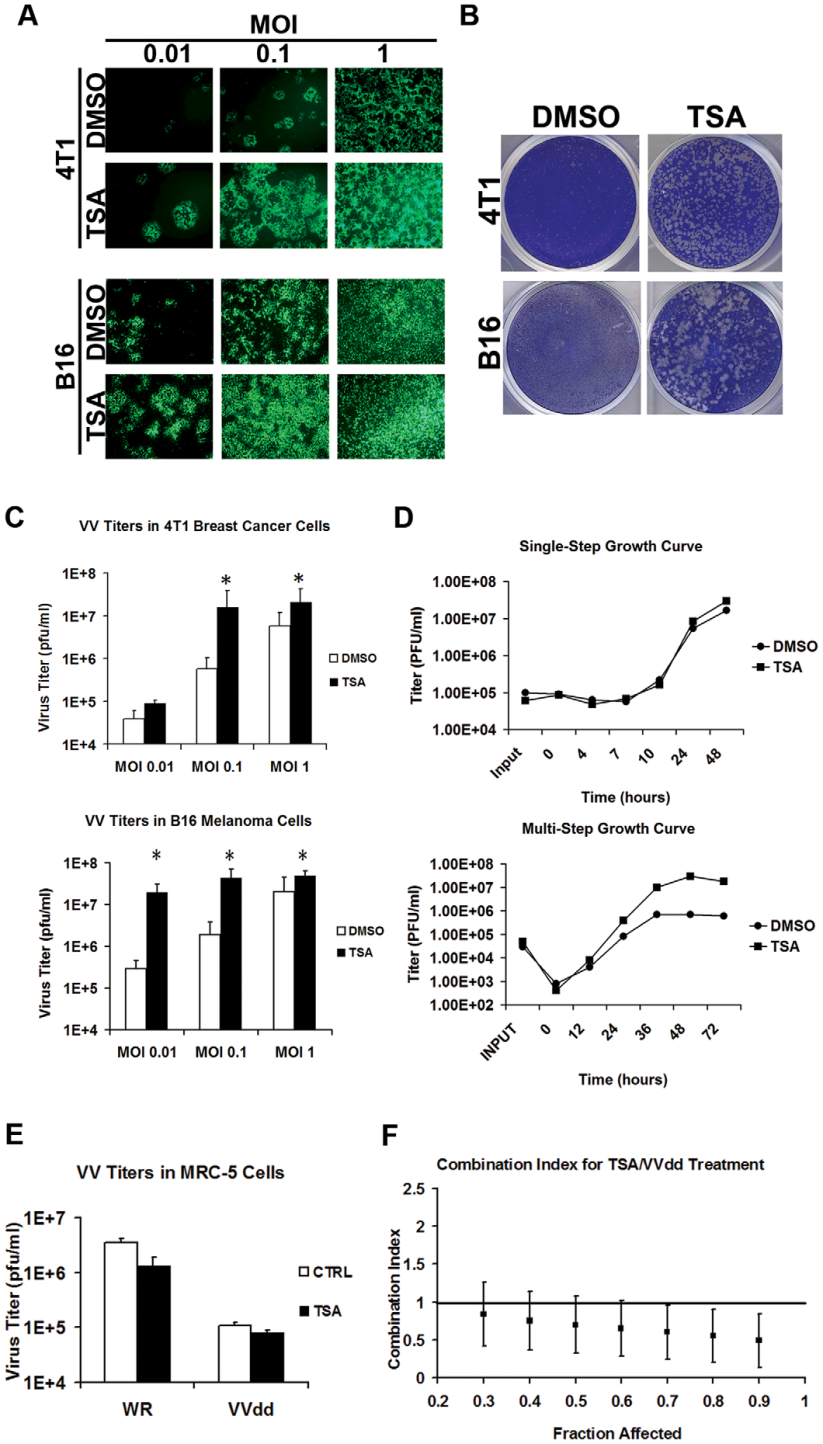
TSA enhances the spread and replication of VVdd *in vitro*. (A) 4T1 breast cancer and B16 melanoma cells were pretreated with TSA (0.0375 uM) for 3 hr and infected at an MOI of 0.01, 0.1 or 1 pfu/cell. Fluorescence microscopy pictures were taken at 72 hours post infection. (B) 4T1 and B16 cells were infected with VVdd at an MOI of 0.1 and 0.01 respectively and plaques were stained for visualization with Coomassie Blue 72 hours post-infection. (C) 4T1 and B16 cells were infected with VVdd-GFP (MOI of 0.01, 0.1, and 1) and samples were collected after 72 hours. Viral titers were measured by standard plaque assay on U2OS cells. The experiment was done in triplicate. Star indicates P value <0.05. (D) B16 cells were pre-treated with TSA 0.0375 µM for 3 hours then challenged with VVdd at an MOI of 3 (Single step growth curve, top panel) or an MOI of 0.1 (Multi-step growth curve, bottom panel). Samples were collected at the indicated times and viral titers assessed by plaque assay on U2OS cells. (E) MRC-5 normal human fibroblasts were plated into 6-well plates and treated with PBS or a non-toxic concentration (1 uM) of TSA for 6 h. At the end of the pre-treatment, cells were infected at an MOI of 1.0 by vaccinia strain VVdd or wild type WR. Cells and media were collected after 72 h and viral pfu/cell titered by plaque assay on BSC-1 cells. (F) B16 cells were pre-treated for 4T1 cells were treated with serial dilutions of a fixed ratio combination mixture of VVdd and TSA (1562 PFU: 1 µM VVdd:TSA). Cytotoxicity was assessed using alamar blue reagent after 96 h. Combination indices (CI) were calculated according to the method of Chou and Talalay using Calcusyn. Plots represent the algebraic estimate of the CI in function of the fraction of cells affected (Fa). Error bars indicate the estimated standard error.

Murine 4T1 breast cancer cells and B16 melanoma cells were plated *in vitro* and confluent cells were pre-treated with low-dose TSA (37.5 nM) before infection with VVdd-GFP at MOIs of 0.01, 0.1 and 1 pfu/cell. Figure 1a shows that TSA enhanced VVdd-associated GFP expression in both murine cancer cell lines. TSA treatment resulted in visibly more and larger plaques (Figures 1a and b) and increased viral titers of up to 100-fold (Figure 1c) in tumour cells. Performing single-step and multi-step growth curves (starting MOI = 0.1 and 3 respectively) further revealed that TSA-enhanced VVdd growth was more substantial when the virus was provided at a low MOI and allowed to spread (Figure 1d). In earlier studies we and others, demonstrated that VVdd presents attenuated growth in normal cells when compared to the wild type Western Reserve strain [12]. In figure 1e it is evident that the attenuated growth of VVdd in normal MRC-5 fibroblasts is not affected by treatment with TSA. Combination indices calculated as described by Chou and Talalay [25] revealed that TSA and VVdd combination therapy results in synergistic killing in 4T1 cells (Figure 1f).

### TSA enhances VVdd efficacy in a syngeneic lung metastasis model

**Figure 2:**
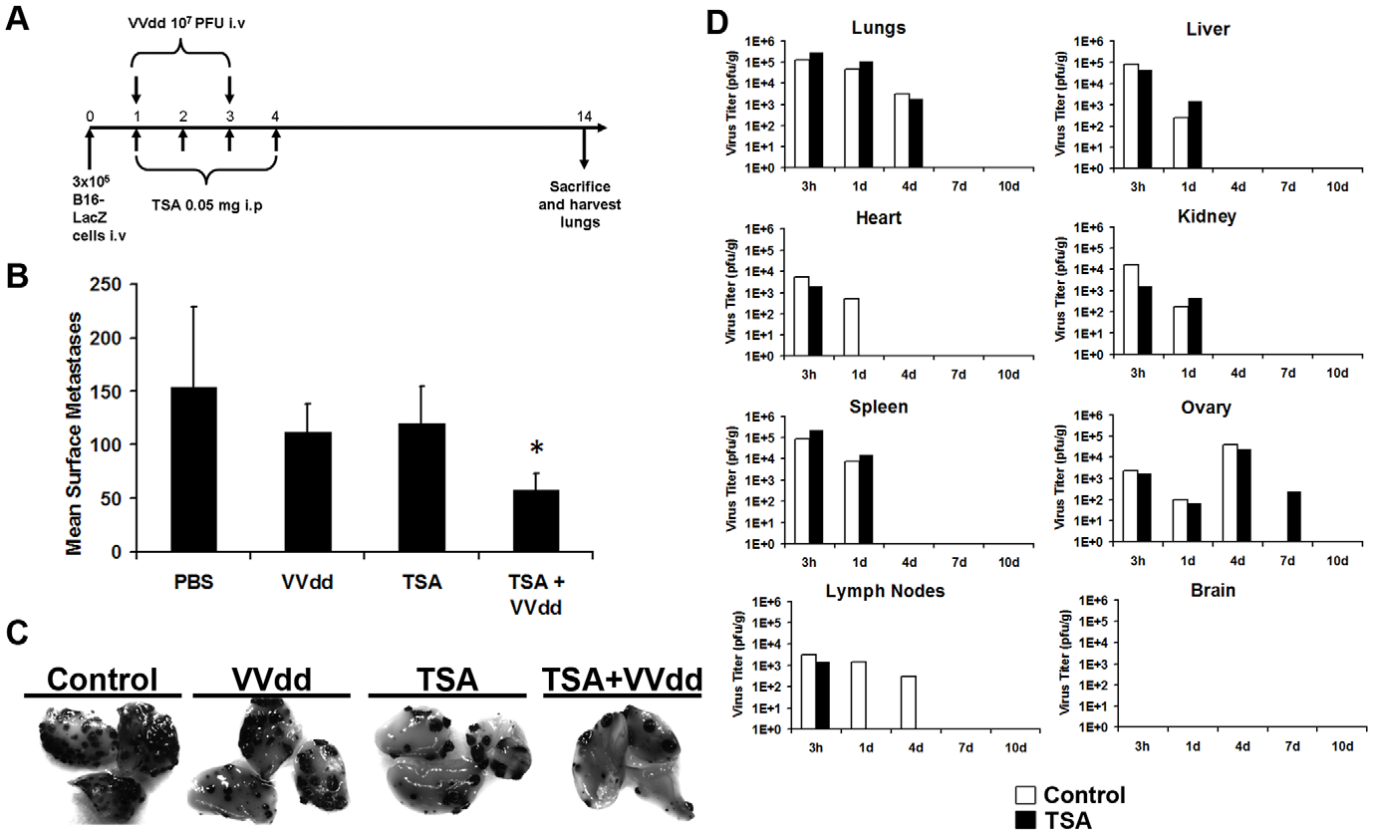
TSA is an effective enhancer of vaccinia *in vivo*. (A) C57BL/6 mice were injected intravenously with B16F10LacZ melanoma cells (3×10^5^ cells/mouse) and TSA (4 daily intraperitoneal doses of 0.05 mg/mouse) alone or in combination with VVdd (1×10^7^ pfu/mouse). (B) The mice were sacrificed after 14 days and the lung metastases were counted after staining with X-gal. Asterix * indicates a P value <0.05 and is significantly different than PBS group and each of the single treatment groups. (C) Lung lobes from control or VVdd and TSA treated mice. B16F10LacZ cells stained with X-Gal. (D) Balb/C mice pre-treated or not with TSA (0.05 mg/mouse) on days 0 through 3. After 3 hr pre-treatment on day 0, mice were given an intra-venous dose of VVdd-luciferase of 1×10^8^ pfu/mouse. One mouse per group was sacrificed at each time point and organs were titered for virus content by standard plaque assay on U2OS cells.

We next tested the ability of TSA to enhance VVdd activity in a mouse tumour model. To this end, C57/Bl6 mice were injected intravenously with 3×10^5^ B16F10LacZ melanoma cells. Following tumour seeding in the lungs, mice were treated with either TSA (4 daily intraperitoneal doses of 0.05 mg) and/or VVdd (2 intravenous injections of 1×10^7^ pfu/mouse). This treatment schedule can be visualized in figure 2a. At the doses used, mice did not display any discernable side effects from treatment with TSA, VVdd, or the combination of both. Two weeks following implantation, lungs were collected and tumours identified (figure 2c) by using established X-gal staining procedures [26], [27]. As single agents at the doses used, both TSA and VVdd reduced the number of lung tumours in treated animals compared to vehicle treated controls. When used in combination however a further therapeutic benefit was obvious with significantly fewer lung metastases observed than with either agent alone (Figure 2b, p<0.05). To determine if the effect of TSA was limited to VVdd growth in tumour tissues, we carried out bio-distribution studies in a sampling of normal tissues in infected animals. At the indicated times (figure 2d) infected animals were sacrificed and virus titers in selected organs determined by plaque assay. We found that treatment with TSA did not generally lead to increased VVdd replication in normal tissues, although clearance of VVdd appeared to be slightly quicker in lymph nodes and heart while prolonged in ovaries. In light of this, we further tested the effect of TSA on VVdd growth in mouse lymph node, heart and ovarian tissue explants as well as three normal primary ovarian cell lines. We found that TSA treatment did not significantly change virus yields from tissue explants of any origin, not did it increase titers obtained from normal ovarian cells (Supplemental figure S1a-c). These findings coupled with the increased anti-tumour activity and minimal toxicity suggests the TSA/VVdd combination therapy may be clinically beneficial.

### TSA enhances an attenuated B18R-deleted vaccinia strain

**Figure 3:**
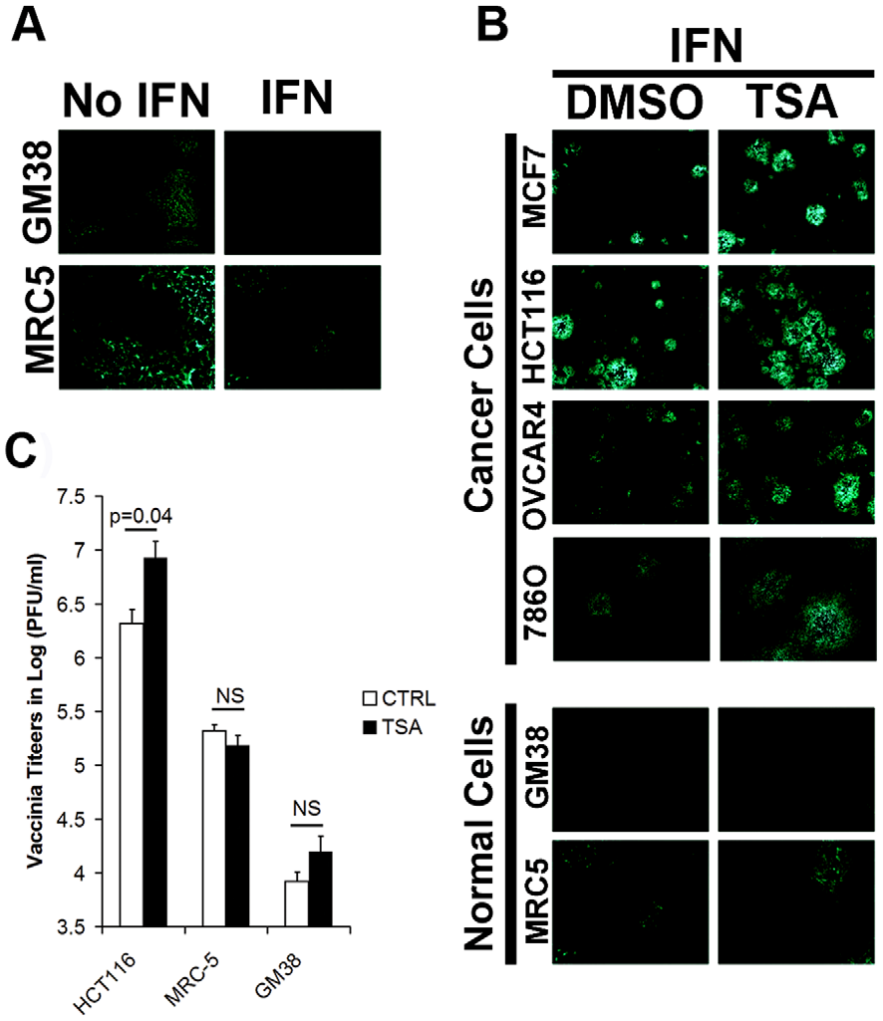
TSA enhances vaccinia in presence of IFN in cancer cells. (A) Normal cells GM38 and MRC-5 were treated or not with 200 IU/ml of IFN for 16 hrs. Cells were then infected with VVdd at MOI 0.001 and fluorescent pictures were taken after 72 h. (B) Cells were pre-treated for 3 h with TSA (0.04 µM) and then 200 IU/ml of IFN for 16 h. Cells were subsequently infected with VVdd at MOI 0.001 and fluorescent pictures were taken after 72 h. (C) Cells were treated as in (B) but samples were collected after 72 h for tittering on U2OS cells. Error bars indicate the standard error. NS stands for non-significant (n = 3, ANOVA).

Although vaccinia encodes a number of interferon signaling antagonists [15], [16], [17], [28], [29], [30], it nonetheless can have its growth attenuated by pre-treatment of normal cells with interferon (IFN) prior to the time when virus can initiate the production of its armament of innate immune suppressing proteins. As shown in Figure 3a, pre-treatment of normal human fibroblast GM38 and MRC-5 cells with IFN led to strong inhibition of virus replication and spread. Importantly, the protective effect of IFN was not overcome by TSA in these same cells (Figures 3a, c). In contrast, TSA enhanced VVdd spread as evidenced by vaccinia-associated GFP expression in several cancer cell lines even in the presence of IFN (Figure 3b). In the HCT116 cell line, we further confirmed that this leads to significant increases in viral titers as expected (Figure 3c). These results demonstrate that TSA can enhance vaccinia spread even in the presence of IFN in cancer cells but that TSA cannot overcome an IFN-induced anti-viral state in normal cells.

**Figure 4:**
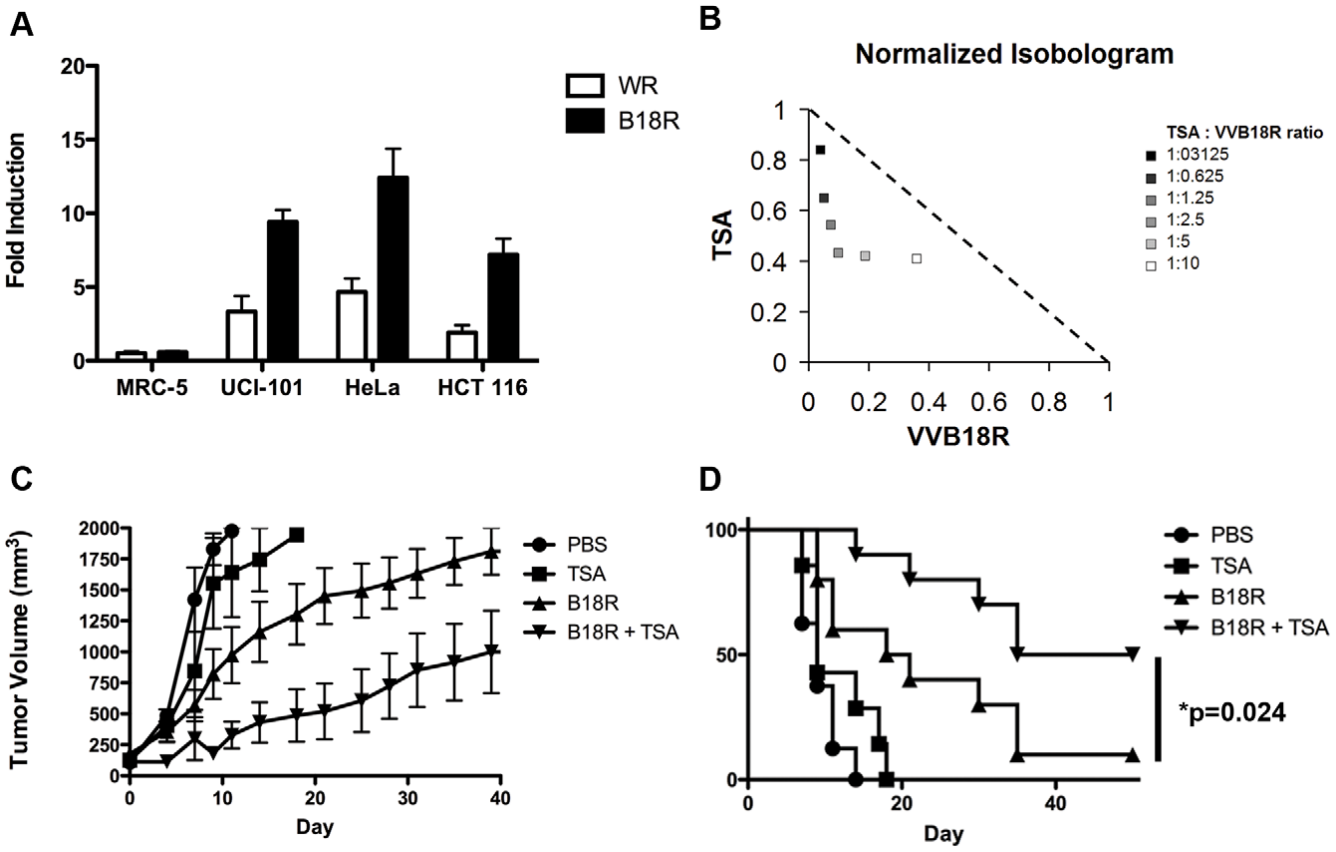
TSA enhances B18R-deleted vaccinia *in vitro* and increases survival in a xenograft model. (A) Human cell lines (MRC-5; UCI-101, HeLa or HCT116) treated with PBS or TSA (1 µM) for 6 h. At the end of the pre-treatment, cells were infected at an MOI or 1.0 by vaccinia strains Western Reserve or Western Reserve with a deletion in the B18R gene. Cells and media were collected after 72 h and viral pfu/cell titered by plaque assay on BSC-1 cells. Fold induction of titer upon TSA treatment relative to control is reported. (B) HCT116 cells were plated in 96-well plates and pre-treated with 0.1 µM TSA for 3 hours then infected with B18R-deleted vaccinia virus at varying MOIs, resulting in the TSA: VVB18R ratios indicated in the figure legend. Cytotoxicity data was obtained using alamar blue 72 hours later and data was analyzed using Calcusyn according to the Chou and Talaly method and the resulting isobologram was plotted. The fraction of the EC_50_ doses for each VVB18R and TSA required in order to result in 50% cell killing for the combination are drawn on the x-axis and y-axis, respectively. Notably, if the combination data point falls on the diagonal (dashed line), an additive effect is indicated; if it falls on the lower left, synergism is indicated; and if falls on the upper right, antagonism is indicated. (C–D) Athymic nu-/nu- mice were implanted subcutaneously with HCT-116 cells (5×10^6^ cells/mouse). Once palpable tumours had formed (50–100 mm^3^), mice were treated with either (i) intraperitoneal PBS; (ii) intraperitoneal TSA (6 µg/mouse) on days −1, 0 and 2; (ii) intravenous injection of WR B18R− TK− Luc+ (1×10^8^ pfu/mouse) on day 0; or (iv) both TSA and WR B18R− TK− Luc+ (n = 10 mice/group). Subsequent tumour burden was followed by caliper measurement (C) and mice were sacrificed when their tumours reached 1400 mm^3^. Percent survival of mice is graphed (D, p = 0.024).

Since the vaccinia virus B18 protein (encoded by the B18R gene) plays a major role in scavenging IFN secreted from infected cells [15], [16], [17], we predicted that TSA may be particularly effective in enhancing the growth of vaccinia strains in which the B18R gene has been deleted [18]. We have previously created an oncolytic version of vaccinia virus by deletion of the B18R gene and while as predicted, this virus replicates poorly in normal cells it still replicates in and kills a wide spectrum of cancer cells [18]. However in tumour cells that retain some interferon responsiveness the B18R deleted virus is less effective than a B18R-replete virus. To investigate the growth of a B18R deleted vaccinia in combination with TSA, the fold induction in plaque forming units produced was compared between TK-deleted and TK/B18R-deleted WR strains in several cell lines. Whereas TSA did not lead to any increase in viral production in normal MRC-5 cells for either strain, it increased viral titers for both strains in human UCI-101 ovarian cancer cells, HeLa cervical cancer cells, and HCT116 colon cancer cells (Figure 4a). In these three cancer cell lines the growth of B18R deleted virus is significantly less than WR parental strain however this deficit can be overcome by incubation of infected cells with TSA. Thus the relative fold induction of B18R deleted virus by TSA is significantly higher than in WR infected cells (figure 4a). This TSA-induced increase in B18R-deleted virus replication led to synergistic cytotoxicty in HCT116 cells as determined by isobologram analysis (Figure 4b). These results support the idea that the spectrum of cancer cells a B18R deleted virus can effectively destroy can be enhanced with TSA without compromising the superior safety of this oncolytic virus, as evidenced from viral bio-distribution studies done for this virus in control conditions or in presence of TSA (supplemental Figure S2a). We further tested the possibility that the combination of B18R-deleted vaccinia virus and TSA could be effective in a human xenograft tumour model. Immunocompromised mice with palpable HCT-116 colon cancer tumours were treated with TSA (or vehicle) and a luciferase-expressing B18R/TK-deleted virus and IVIS imaging was used to examine the growth of the virus in tumour bearing mice. Treatment with TSA resulted in increased virus-associated luciferase activity within HCT-116 tumours when compared to treatment with vaccinia virus alone (Supplemental Figure S2b). The low virus signal in the lungs after 48 h is consistent with the biodistribution data; however this signal is gone by 4 days. Importantly the signal in the lungs is not enhanced by TSA treatment whereas the signal is greatly enhanced in the tumour. Consistent with this observation and the results obtained in the lung metastasis model (Figure 2a), mice treated with the combination of TSA and TK/B18R-deleted WR had delayed tumor progression (Figure 4c) and demonstrated increased survival versus mice treated with either agent alone (Figure 4d, p = 0.024).

## Discussion

Trichostatin A (TSA) was one of the first HDAC inhibitors to be discovered and although its anti-cancer properties are well documented, its sub-optimal *in vivo* stability has made it less attractive for use as a chronically administered anti-cancer drug [31], [32]. A considerable effort in the HDI field has led to the development of more stable TSA derivatives such as Vorinistat ® (SAHA), which was recently approved for limited applications like treatment of CTCL [33], [34], [35], [36]. In this study we found that SAHA was significantly less effective at augmenting vaccinia virus spread *in vitro* than TSA. In this context, we also found that TSA interacted synergistically with vaccinia virus, leading to better cell killing. Theoretically, this synergistic interaction predicts that the effective dose used for each therapeutic *in vivo* could be reduced while retaining efficient anti-tumour activity. However, since OVs need to overcome numerous physiological barriers in order to reach tumors, it is likely that TSA/Vaccinia combination therapy would be best used as a means to increase efficacy as opposed to dose reduction. Nonetheless, this suggests that the relatively short half-life of TSA *in vivo* may not be a concern for the therapeutic application described here in light of the relative potency of its vaccinia-enhancing effect.

Oncolytic virus therapy is an acute treatment with curative intent. Indeed the activity of OVs involves not only replication in, and destruction of tumour cells but also the recruitment of host immune cells to the tumour bed leading to the initiation of anti-tumour immunity. It is known that HDIs can impact the patient's immune cells [37], [38], [39] and thus a fast acting, virus enhancing, compound that is rapidly cleared once an infection is established may be preferred. Furthermore the short half-life of TSA allows for better control over the OV dose should treatment need to be stopped abruptly. Given that our data shows anti-tumoral activity of several different VV strains can be enhanced by TSA *in vivo* the clinical application of TSA may need to be re-visited. This is of considerable interest since VV strains such as JX-594 (Wyeth TK- deleted, GM-CSF expressing) and JX-929 (Western Reserve TK/VGF-deleted, GM-CSF expressing) are currently undergoing Phase I/II clinical trials.

The effect of TSA on the IFN response is well documented [40], [41] and the enhancing effect of HDIs such as TSA on IFN-sensitive strains including VSV and HSV has been previously reported [22]. It is therefore not surprising that TSA can increase the activity of B18R-deleted VV strains. However our finding that vaccinia with an intact B18R gene is still enhanced by TSA suggests that either the anti-interferon activities of B18R are insufficient to completely impair the cellular interferon response or alternatively the effects of TSA go beyond interferon induced anti-viral responses. While we cannot distinguish between these possibilities at the present time, the latter explanation seems likely to us since we and others have found using microarray analysis of HDI treated cells, that hundreds of cellular transcripts induced by viral infection are affected by blocking histone deacetylase activity [23], [42].

Using small molecule OV-enhancers is attractive from a clinical standpoint. In principle, this strategy allows for quite significant genetic attenuation of OVs to restrict growth in normal tissues with conditional rescue of the virus replication in tumour cells following treatment with an enhancing compound like an HDI or other classes of molecules that can complement viral defects [42]. In this and our previous studies, one of the key features of small molecule complementation of attenuated viruses is that the effect of virus enhancers is restricted to tumour cells with minimal impact on the anti-viral programs of normal tissues. This clearly is the case for TSA in the studies presented here where we demonstrate both *in vitro* and in animal models that TSA does not enhance virus growth in normal tissues. The reason for this selectivity is unclear at this time however tumour cells are known to have elevated levels of histone deacetylases suggesting that perhaps malignancies have evolved more dependency upon this type of epigenetic modification to control gene expression [43]. Alternatively since tumour cells have often inactivated at least some components of their anti-viral programs, it may be that the addition of HDIs simply “break the camel's back” in tumour cells but are ineffective in normal tissues that have fully intact multi-layered protection systems. Further studies are currently under way to address some of these issues.

We conclude that TSA is a potent enhancer of VV *in vitro* and *in vivo.* We propose that HDIs such as TSA could be used to enhance the effectiveness of OVs *in vivo* and that further clinical evaluation of this possibility is warranted.

## Materials and Methods

### Cells and Viruses

4T1 murine breast cancer cells, HCT116, MCF7, OVCAR4, 786O and MRC5 cells were obtained from ATCC. Maintained in Dulbecco's modified Eagle's medium (DMEM) supplemented with 10% 3∶1 calf serum: fetal bovine serum and grown at 37°C with 5% CO_2_. B16F10-LacZ murine melanoma cells were obtained from ATCC and maintained in αMEM supplemented with 10% 3∶1 calf serum: fetal bovine serum. GM38 cells were kindly provided by Dr. Bruce McKay (Ottawa Hospital Research Institute, Ottawa, ON) and maintained in DMEM supplemented with 15% 3∶1 calf serum: fetal bovine serum. Human lines MRC-5, UCI-101 and HeLa were provided by Stephen Thorne. NOV2963D, NOV3128D and NOV3198G normal ovarian cell lines were kindly provided by Dr. Anne-Marie Mes-Masson (Institut du Cancer de Montreal, Montreal, QC, Canada) and were grown in OSE (Wisent, QC, CA) media supplemented with 10 ng/ml endothelial growth factor, 34 µg/ml bovine pituitary extract, 5 µg/ml insulin and 0.5 µg/ml hydrocortisone. VVdd was derived from the wild type strain Western Reserve with a double deletion of the genes thymidine kinase and vaccinia growth factor [12]. Green fluorescent protein was inserted at the TK locus. Virus was propagated in U2OS cells. Lastly, the wild type Western Reserve (WR) and WR B18R-TK-Luc+ were also used in *in vitro* and *in vivo* experiments.

### Fluorescence Microscopy and Fluorescence Quantification

A fluorescent microscope (Zeiss Axiovert S 100) was used to photograph the cells. The GFP expressing virus can be visualized with a fluorescence microscope. Infected cells with actively replicating virus appear green under the fluorescent microscope. Images were quantified for green fluorescence using the image analysis software Image J (NIH).

### In Vitro Assays and Cell Staining

The HDI screening was done in 96-well plates with 20,000 cells per well. Cells were plated and 24 hours later were pre-treated for 3 hours with indicated HDI. The drugs: SAHA (Exclusive Chemistry, Obninsk, Russia), MS-275 (Selleck Chemicals, Houston, TX, USA), Oxamflatin (Alexis Biochemicals, Plymouth Meeting, PA, USA), Apicidin (Alexis Biochemicals, Plymouth Meeting, PA, USA), SBHA (Enzo Life Sciences International Inc., Plymouth Meeting, PA, USA), Scriptaid (Sigma), Valproic acid (Sigma), CHAHA (Sigma), M344 (Sigma) and Trichostatin A (Sigma) were added to wells at indicated concentrations and cells were infected with VV. Images spanning well surface were quantified as described above. For further TSA testing *in vitro* cells were plated in 12 well plates with 2.5×10^5^ cells per well. Once confluence was reached, the cells were pre-treated with TSA (0.0375 µM) and DMSO as the drug vehicle control. After 3 hours of pre-treatment, the virus was added at the indicated multiplicity of infection. Fluorescence images were taken after 24, 48 and 72 hours. Cells were collected after 72 hours and frozen at −80°C for titering on U2OS cells. Other wells were stained for plaques by first rinsing each well with PBS, then fixing the cells for 10 minutes using 3∶1 ratio of methanol to acetic acid. After the cells were fixed, they were stained with Coomassie Blue to visualize viral plaques. Cell lines GM38, MRC5, HCT116, MCF7, 786O and OVCAR4 were pre-treated for 3 hrs with TSA and then treated with 200 IU/ml of IFN (Intron A from Schering, Kenilworth, NJ) overnight (16 hrs) and then infected with vaccinia at various MOIs. For combination index 20,000 4T1 cells were plated in 96-well plates. Cells were treated with serial dilutions of a fixed ratio combination mixture of VVdd and TSA (1562 PFU: 1 µM VVdd:TSA). Alternately in Figure 4a, HCT116 cells were plated similarly but treated with drug:virus at the indicated ratio, where the TSA concentration was 0.1 µM. Cytotoxicity was assessed using alamar blue reagent after 96 h. Combination indices (CI) were calculated according to the method of Chou and Talalay using Calcusyn [25]. In Figure 1f, Plots represent the algebraic estimate of the CI in function of the fraction of cells affected (Fa). Error bars indicate the estimated standard error.

### Titration of virus samples

Each sample (cells and supernatants) was collected from the well and cells were lysed by freezing and thawing (−80°C to 37°C) three times. Samples were diluted serially by a factor of 10 and 500 µL of each dilution were put on confluent U2OS or BSC-1 cells in a 12 well plate (5×10^5^ cells per well plated 24 hours prior). The samples are placed in an incubator for 2 hours at 37°C to allow the virus to enter the cells. After the 2 hour incubation, the virus sample was removed from the U2OS cells and an 1 mL of an overlay solution was put on the cells (1∶1 ratio of 3% CMC: 2XDMEM +20% FBS). The plates were left to incubate at 37°C for 72 hours. After 72 hours, the overlay was removed and each well was stained with Coomassie Blue to visualize and count the plaques (see above for staining).

### Western Blot

4T1 cells were plated in 10 cm dishes and treated with HDI at the indicated concentrations. The following day cells were lysed with Radioimmunoprecipitation assay (RIPA) lysis buffer containing protease inhibitor (Sigma, P2714). Equal amounts of proteins collected from samples were electrophoresed on a 15% SDS-polyacrylamide gel. Gels were blotted on nitrocellulose membranes and detected by Western blot analysis probing with the antibody Anti-hyperacetylated Histone H4 (Penta, 06-946) diluted 1∶5,000. Actin was used as the loading control and was detected by mouse actin antibody (Sigma) diluted 1∶10,000.

### Lung metastasis model

B16F10 LacZ cells were injected intravenously into female C57BL/6 mice from Charles River Laboratories (Wilmington, MA). Each mouse was injected with 3×10^5^ cells into the tail vein on day 0. On days 1–4 mice were given intra-peritoneal injections of TSA of 0.05 mg/mouse. On days 1 and 3 mice were given 1×10^7^ pfu/mouse of VVdd intravenously via the tail vein. On day 14 mice were sacrificed and lungs were dissected. Lung tumours were stained with the substrate X-gal and each metastasis was counted [26], [27].

### Colon Cancer Survival Model

Athymic nu-/nu- mice were implanted subcutaneously with HCT-116 cells (5×10^6^ cells/mouse). Once palpable tumours had formed (50–100 mm^3^), mice were treated with either (i) intraperitoneal PBS; (ii) intraperitoneal TSA (6 µg/mouse) on days −1, 0 and 2; (iii) intravenous injection of WR B18R- TK- Luc+ (1×10^8^ pfu/mouse) on day 0; or (iv) both TSA and WR B18R- TK- Luc+ (n = 10 mice/group). Subsequent tumour burden was followed by caliper measurement and mice sacrificed when their tumours reached 1400 mm^3^. In addition, mice receiving viral treatment were imaged by bioluminescence imaging at regular times after treatment to assess viral gene expression. Mice were injected with D-luciferin (Molecular Imaging Products, Ann Arbor, MI) for firefly luciferase imaging. Mice were anesthetized under 3% isoflurane (Baxter, Deerfield, IL) and imaged with (IVIS200, Xenogen, part of Caliper Life Sciences). Data acquisition and analysis were performed using Living Image v2.5 software.

### Biodistribution

Balb/C mice were pre-treated with 0.05 mg of TSA per mouse or a control by intra-peritoneal injections on day 0. Mice were given the same dose of TSA or control each day for days 0 through 3. Mice were give 1×10^8^ pfu/mouse of VVdd-luciferase or luciferase-expressing B18R/tk-deleted vaccinia virus by intravenous injection into the tail vein on day 1 after a 3 hour pre-treatment with TSA. One mouse from each condition (treated or not with TSA) was sacrificed at various time points. Mice were sacrificed 3 hours after virus injection and on day 1, 4, 7 and 10. The following organs were collected for titering: lymph nodes (brachial and inguinal), ovaries, spleen, kidney, liver, lungs, heart and brain. Organs were homogenized in PBS and titered by standard plaque assay on U2OS cells (see above).

## Supporting Information

Figure S1 TSA does not increase in vitro infection of normal mouse tissues or human normal ovarian primary cell lines. (A) Mouse Lymph nodes (LN), Heart, and Ovaries were obtained by dissection and immediately put in cell culture. Tissues were subsequently pretreated with 0.1 µM TSA for 24 hours and infected with 1E7 PFU VVdd-GFP. 72 hours later, tissues were collected, weighed, and homogenized in PBS using a tissue homogenizer. Homogenates were subsequently titered on U2OS cells and VVdd PFU/g of tissue was graphed. (B) Normal human ovarian primary cells (NOV2963D, NOV3128D, and NOV3198G) were plated in 96-well plates (25 000 cells/well) overnight and pre-treated with TSA 0.04 µM for 3 hours. Subsequently, cells were infected with VVdd-GFP at an MOI of 0.001. 72 hours later, pictures were taken by fluorescence microscopy. Cells and supernatant were subsequently harvested and titered on U2OS cells. Titers are presented in (C). Error bars represent the standard error. NS stands for non significant (ANOVA, n = 3).
(1.68 MB TIF)

Figure S2 TSA increases virus-associated luciferase activity in subcutaneous tumour. (A) Balb/C mice pre-treated or not with TSA (0.05 mg/mouse) on days 0 through 3. After 3 hr pre-treatment on day 0, mice were given an intra-venous dose of B18R-deleted at 1×10^8^ pfu/mouse. One mouse per group was sacrificed at each time point and organs were titered for virus content by standard plaque assay on U2OS cells. (B) Athymic nu-/nu- mice were implanted subcutaneously with HCT-116 cells (5×10^6^ cells/mouse). Once palpable tumours had formed (50–100 mm^3^), mice were treated with either (i) intraperitoneal PBS; (ii) intraperitoneal TSA (6 µg/mouse) on days −1, 0 and 2; (ii) intravenous injection of WR B18R− TK− Luc+ (1×108 pfu/mouse) on day 0; or (iv) both TSA and WR B18R− TK− Luc+ (n = 10 mice/group). Viral replication at tumour sites was imaged using in vivo imaging system 48 hours after virus injection for luciferase.
(1.46 MB TIF)
