# Supplementary figures and images for: Enhancement of Vaccinia Virus Based Oncolysis with Histone Deacetylase Inhibitors
Source: PLoS One. 2010 Dec 30;5(12):e14462. doi: 10.1371/journal.pone.0014462 (PMC3012680; doi:10.1371/journal.pone.0014462)

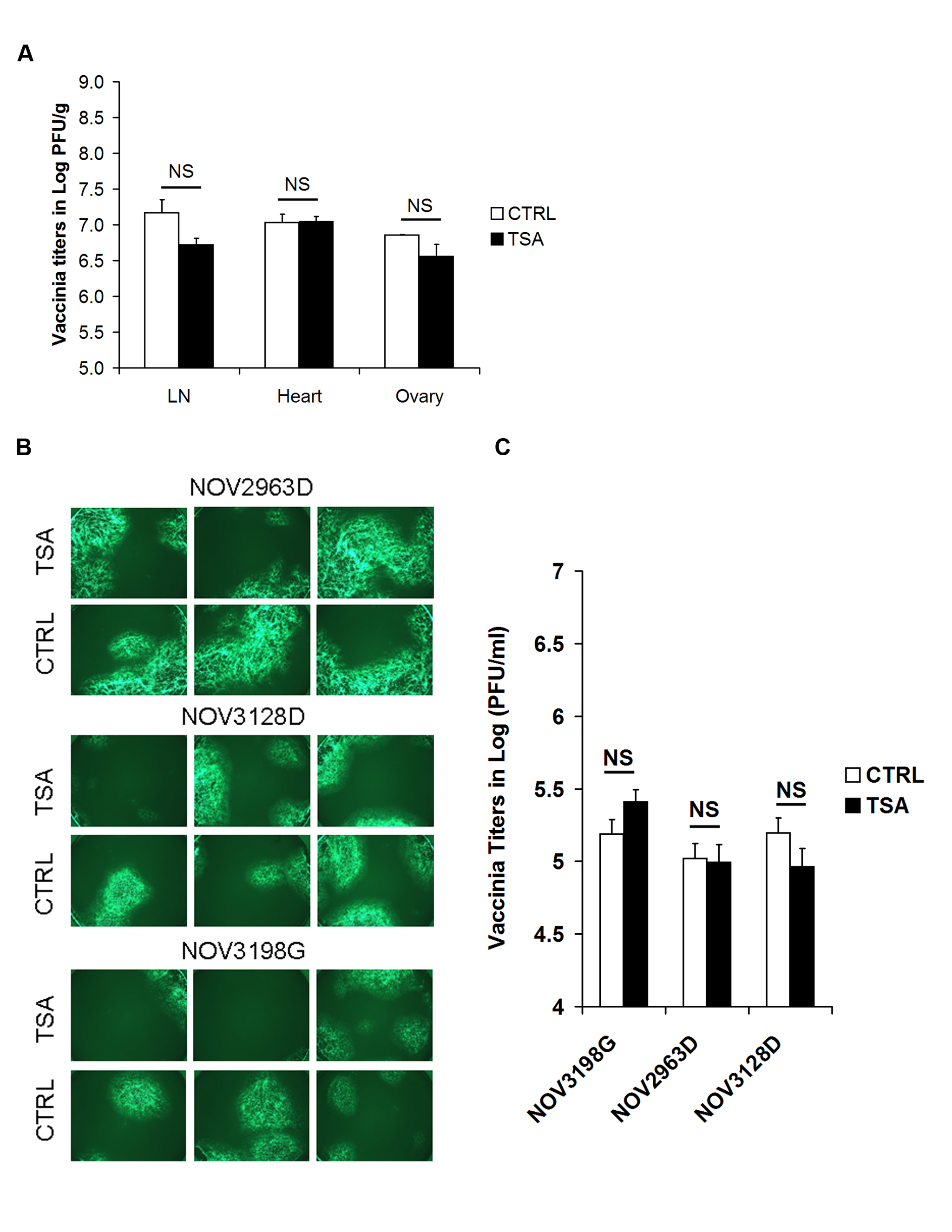

Supplement: Figure S1 — TSA does not increase in vitro infection of normal mouse tissues or human normal ovarian primary cell lines. (A) Mouse Lymph nodes (LN), Heart, and Ovaries were obtained by dissection and immediately put in cell culture. Tissues were subsequently pretreated with 0.1 µM TSA for 24 hours and infected with 1E7 PFU VVdd-GFP. 72 hours later, tissues were collected, weighed, and homogenized in PBS using a tissue homogenizer. Homogenates were subsequently titered on U2OS cells and VVdd PFU/g of tissue was graphed. (B) Normal human ovarian primary cells (NOV2963D, NOV3128D, and NOV3198G) were plated in 96-well plates (25 000 cells/well) overnight and pre-treated with TSA 0.04 µM for 3 hours. Subsequently, cells were infected with VVdd-GFP at an MOI of 0.001. 72 hours later, pictures were taken by fluorescence microscopy. Cells and supernatant were subsequently harvested and titered on U2OS cells. Titers are presented in (C). Error bars represent the standard error. NS stands for non significant (ANOVA, n = 3). (1.68 MB TIF) [file pone.0014462.s001.tif]

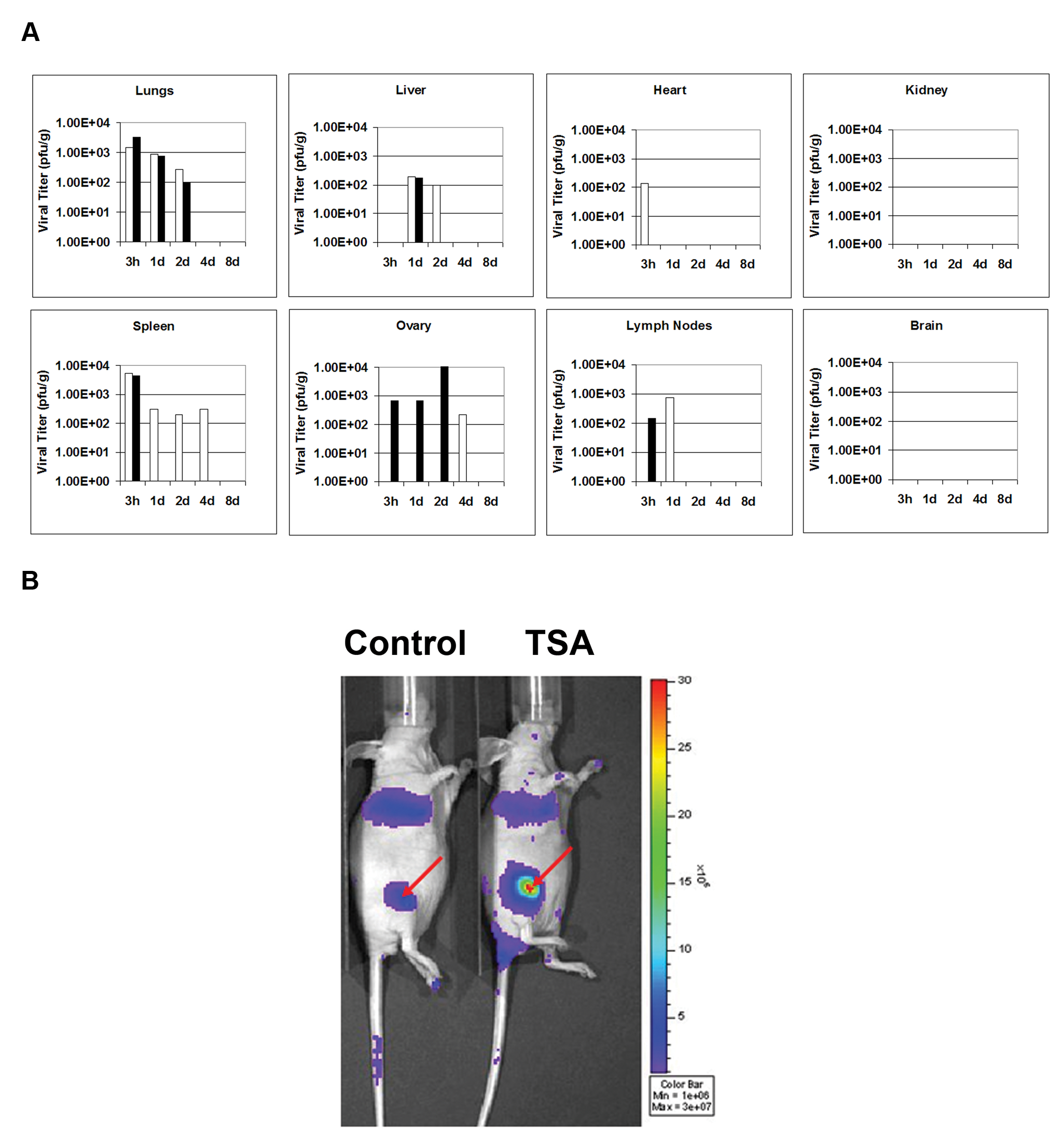

Supplement: Figure S2 — TSA increases virus-associated luciferase activity in subcutaneous tumour. (A) Balb/C mice pre-treated or not with TSA (0.05 mg/mouse) on days 0 through 3. After 3 hr pre-treatment on day 0, mice were given an intra-venous dose of B18R-deleted at 1×10^8^ pfu/mouse. One mouse per group was sacrificed at each time point and organs were titered for virus content by standard plaque assay on U2OS cells. (B) Athymic nu-/nu- mice were implanted subcutaneously with HCT-116 cells (5×10^6^ cells/mouse). Once palpable tumours had formed (50–100 mm3), mice were treated with either (i) intraperitoneal PBS; (ii) intraperitoneal TSA (6 µg/mouse) on days −1, 0 and 2; (ii) intravenous injection of WR B18R− TK− Luc+ (1×10^8^ pfu/mouse) on day 0; or (iv) both TSA and WR B18R− TK− Luc+ (n = 10 mice/group). Viral replication at tumour sites was imaged using in vivo imaging system 48 hours after virus injection for luciferase. (1.46 MB TIF) [file pone.0014462.s002.tif]
